# Carbon–carbon bond activation by Mg, Al, and Zn complexes

**DOI:** 10.1039/d3sc03336h

**Published:** 2023-09-14

**Authors:** Joseph M. Parr, Mark R. Crimmin

**Affiliations:** a Department of Chemistry, Molecular Science Research Hub, Imperial College London 82 Wood Lane, White City London W12 0BZ UK m.crimmin@imperial.ac.uk

## Abstract

Examples of carbon–carbon bond activation reactions at Mg, Al, and Zn are described in this review. Several distinct mechanisms for C–C bond activation at these metals have been proposed, with the key C–C bond activation step occurring by (i) α-alkyl elimination, (ii) β-alkyl elimination, (iii) oxidative addition, or (iv) an electrocyclic reaction. Many of the known pathways involve an overall 2-electron redox process. Despite this, the direct oxidative addition of C–C bonds to these metals is relatively rare, instead most reactions occur through initial installation of the metal on a hydrocarbon scaffold (*e.g.* by a cycloaddition reaction or hydrometallation) followed by an α-alkyl or β-alkyl elimination step. Emerging applications of Mg, Al, and Zn complexes as catalysts for the functionalisation of C–C bonds are also discussed.

## Abstract 2

Examples of carbon–carbon bond activation reactions at Mg, Al, and Zn are described in this review.

## Graphical abstract

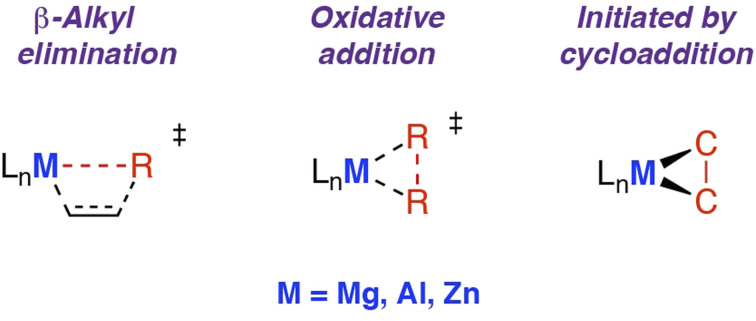

## 1. Introduction

Carbon–carbon (C–C) bonds are ubiquitous, making up the hydrocarbon skeleton of most organic molecules. Reactions that break strong C–C bonds are therefore of broad importance. For example, selective activation of C–C bonds affords a powerful method to alter the hydrocarbon scaffold of molecules.^1,2^ This opens the door for novel synthetic disconnections and routes to complex organic molecules of relevance to medicinal and materials chemistry. Reactions that break C–C bonds also underpin our global energy sector. Globally essential fuels (coal, crude oil, and biomass) are comprised of C–C bonds. Catalytic cracking of C–C bonds in hydrocarbon feedstocks, such as crude oil, converts high molecular weight alkanes to more valuable alkenes and medium-length hydrocarbons (*e.g.* C_7_ to C_9_ alkanes).^3–5^

Reactions that break C–C bonds are challenging to achieve.^6^ C–C bonds are strong and non-polar. To give two examples: (i) ethane, the simplest linear alkane, has a homolytic bond dissociation energy^7^ of 90.1 ± 0.1 kcal mol^−1^, (ii) benzene has a calculated homolytic bond dissociation energy^8^ of 147.0 kcal mol^−1^. The increased thermodynamic stability of C–C bonds in aromatic rings is unsurprising given their π-character and electron delocalisation across the ring. C–C bonds are also sterically protected. They are often buried within the molecular framework and the orbitals involved in bonding are kinetically inaccessible. As such chemoselectivity becomes a key issue, with surrounding C–H bonds often the first sites to react with reagents and catalysts that would otherwise be capable of breaking C–C bonds.^9^

**Scheme 1:**
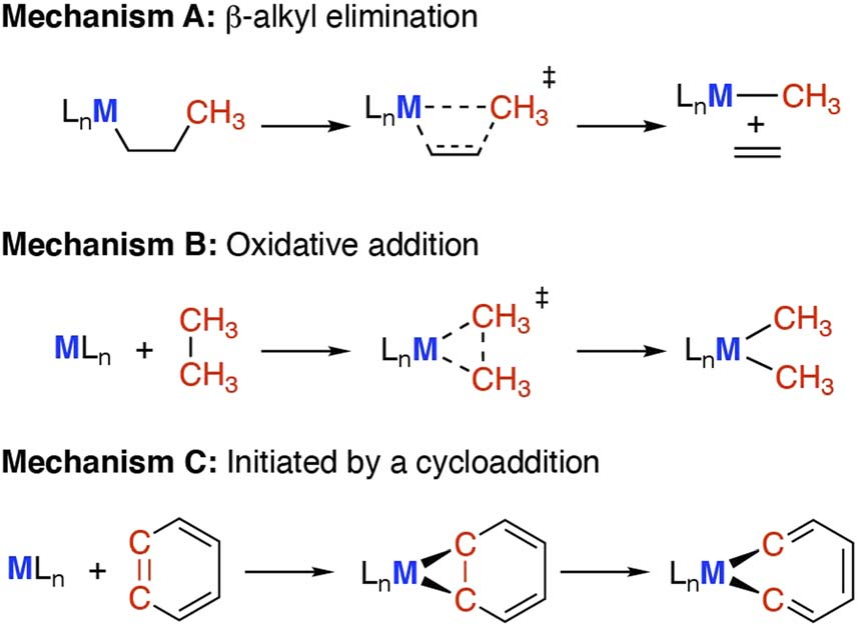
Carbon–carbon bond activation mechanisms, *via* (A) β-alkyl elimination or migration; (B) oxidative addition; (C) cycloaddition reaction and subsequent rearrangement.

Here we define C–C bond activation as a process in which the C–C bond of the σ-framework breaks at a metal centre (M), creating at least one new M–C bond. While C–C bond functionalisation is defined as a process that breaks a C–C bond and transforms into two new C–X bonds (X = H, heteroatom). The activation of C–C bonds has been achieved on the surface of heterogeneous catalysts,^10^ within the active sites of enzymes,^11,12^ and under homogeneous conditions using metal complexes.^13,14^ The systems which are best understood are arguably those that contained well-defined transition metal sites (Co–Ir, Ni–Pt),^15^ where partially occupied valence d-orbitals facilitate C–C bond breaking.^16–18^ Often model substrates that contain weakened C–C bonds and/or extensive ring strain are studied.^19^ For example, hydrocarbons with smaller ring sizes are routinely investigated as the C–C bond strength decreases across the series cyclohexane > cyclopentane > cyclobutane > cyclopropane.^20–22^ Reactivity tends to follow established mechanisms (Scheme 1).

• (A) β-Alkyl elimination, wherein a metal bound alkyl ligand is fragmented into the corresponding metal alkyl and alkene units.^23,24^

• (B) Oxidative addition, wherein the C–C bond is cleaved by addition to a low oxidation state metal complex, increasing the metal oxidation state by two and creating two new M–C bonds.^17^

• (C) Cycloaddition between a hydrocarbon and metal reagent, creating a strained metallocycle which can then undergo C–C bond activation (*e.g.* through α-elimination or an electrocyclic reaction).^25,26^

Though important progress is being made toward transition metal mediated C–C bond activation, there is an increasing drive away from late transition metal-based systems. Late transition metals are commonly expensive and toxic, with further issues regarding the sustainability and ethics of the mining practices used to obtain the requisite minerals for refining.^27,28^ Main-group metals (*e.g.* Mg, Al) along with the post-transition metal Zn are promising alternatives to their transition metal counterparts for applications in synthesis and catalysis. These elements are commonly earth-abundant, inexpensive, and more widely distributed in the Earth's crust compared to the late transition metals.^29^ They are non-toxic and accordingly safer to handle. For some (*e.g.* Al) there are even established networks and processes for recycling, auguring well for a future circular economy.

In this review, we summarise the current examples of C–C bond activation with Mg, Al, and Zn complexes. The scope of the review is limited to these emerging systems with a specific focus on mechanism and understanding. To the best of our knowledge there are no well-defined examples of C–C bond activation with heavier main group (Ca–Ba, Ga–Tl, Pb), or post-transition (Cd–Hg) metals. A limited number of examples of metal free C–C bond activation have been reported for systems using frustrated Lewis pairs,^30^ boron-,^31,32^ silicon-,^33–35^ phosphorous,^36,37^ and organic-compounds.^38^ These examples with non-metals or semi-metals are not the focus of this review and are covered elsewhere.^39^ The discussion is split into three distinct approaches, namely β-alkyl migration, oxidative addition, and those initiated by cycloaddition reactions. Through discussion of mechanism, we aim to highlight the divergent chemistry shown by these complexes compared to their transition metal counterparts and touch on the potential implications in synthesis.

## 2. Carbon–carbon bond activation

### 2.1 β-Alkyl elimination at Mg and Zn metal centres

**Scheme 2:**
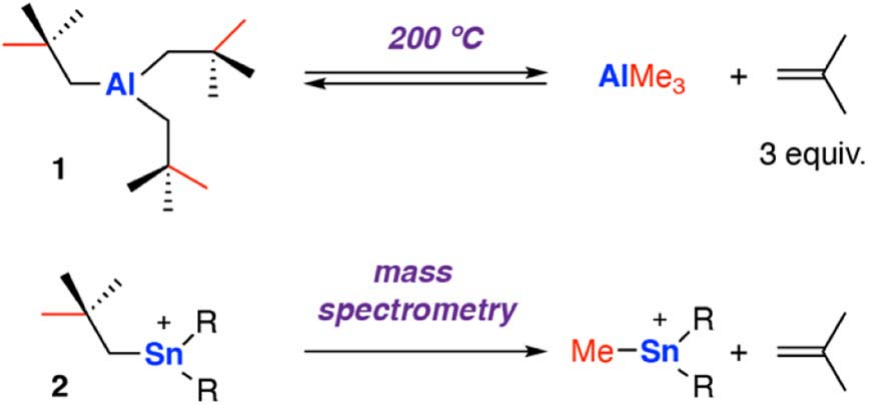
Early examples of β-alkyl elimination at main group metals. The reversible release of iso-butene gas by thermolysis of tris(neo-pentyl)aluminium 1 (top); elimination of iso-butene gas during mass-spectrometer fragmentation of tris(neo-pentyl)stannyl cation 2 (bottom), R = CH_2_CMe_3_.

One of the earliest reports of C–C σ-bond activation by any metal appears to proceed through a β-alkyl migration mechanism at an aluminium centre. In 1960, Pfohl reported the thermolysis of tris(neo-pentyl)aluminium [Al{CH_2_C(Me)_3_}_3_] 1 at high temperature (200 °C).^40^ The reversible stoichiometric formation of iso-butene gas and trimethylaluminium was observed *via* sp^3^ C–C σ-bond activation, presumed to occur through a β-methyl migration reaction (Scheme 2). The release of three equivalents of iso-butene gas provides an entropic driving force for the forward reaction.

In 1999, Dakternieks and co-workers reported a related reaction at a Sn complex, observed during fragmentation in a mass spectrometer.^41^ Application of a high cone voltage (>60 V) to a acetonitrile solution of the tris(neo-pentyl)stannyl cation [Sn{CH_2_C(Me)_3_}_3_]^+^2 showed formation of methyl tin cations and release of isobutene gas. Reaction of the deuterium labelled analog [Sn{CD_2_C(Me)_3_}_3_]^+^ showed the formation of isobutene gas with the alkene protons D-labelled, consistent with a β-alkyl elimination process. An alternate pathway involving homolysis of the Sn–C bond to form a neo-pentyl radical that fragments to iso-butene and a methyl radical that can recombine with Sn, was not ruled out and cannot be discounted under fragmentation conditions in the mass spectrometer.

**Scheme 3:**
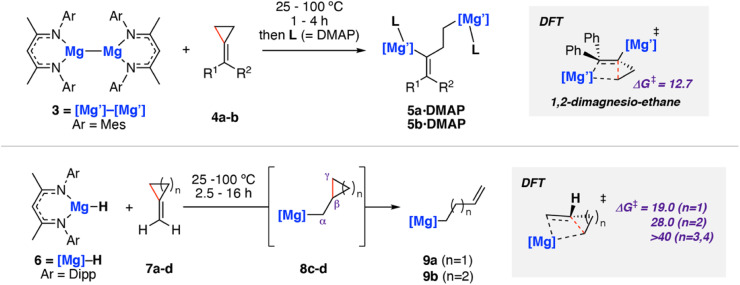
Reaction of magnesium(i) complex 3 with methylidene cycloalkanes 4a–b (top). Reaction of magnesium(ii) hydride complex 6 with 7a–d (bottom). Mes = 2,4,6-trimethylphenyl; Dipp = 2,6-diisopropylphenyl. Energies for Δ*G*^‡^_298 K_ in kcal mol^−1^. 4a (R^1^ = R^2^ = Ph), 4b (R^1^ = Ph, R^2^ = H), 7a–d (*n* = 1–4).

In 2020, we reported C–C σ-bond cleavage of strained alkylidene cyclopropanes using magnesium reagents.^42^ Reaction of the β-diketiminate stabilised magnesium(i) dimer [Mg{CH{C(CH_3_)NMes}_2_}]_2_ (Mes = 2,4,6-trimethylphenyl)^43–46^3 with alkylidene cyclopropanes 4a–b and subsequent addition of dimethylaminopyridine (DMAP) led to the corresponding ring-opened products 5a–b·DMAP (Scheme 3). DMAP was used to trap and help crystallise the products and is not thought to participate in the mechanism of C–C σ-bond activation. DFT calculations support a stepwise mechanism starting with 1,2-addition of the Mg–Mg σ-bond to the alkene.^47^ This step places one of the Mg sites in a suitable position to facilitate β-alkyl migration. β-Alkyl migration from the 1,2-dimagnesio-ethane intermediate is then a relatively facile process (Δ*G*^‡^_298 K_ = 12.7 kcal mol^−1^), yielding the observed products.

This work was extended to include reaction of the related magnesium(ii) hydride complex 6 [Mg(μ-H){CH{C(CH_3_)NDipp}_2_}]_2_ (Dipp = 2,6-diisopropylphenyl) with the same set of substrates (Scheme 3, bottom).^48,49^ Stoichiometric reaction of 6 with methylidene cyclopropane (7a) and methylidene cyclobutane (7b) yielded the ring-opened alkyl magnesium complexes 9a and 9b in good yields. Though no intermediates were observed spectroscopically, DFT calculations and related literature^23,50,51^ supported a hydromagnesiated intermediate 8a–b as a prerequisite to β-alkyl elimination and thus C–C σ-bond activation. Additional evidence for the hydromagnesiated intermediates was gathered through reaction of unstrained methylidene cyclopentane (7c) and methylidene cyclohexane (7d) with 6, which formed the hydromagnesiated products 8c–d in high yields.^52^ Calculated activation barriers for σ-bond C–C bond cleavage for five and six-membered rings were unfeasible under the reaction conditions (Δ*G*^‡^_298 K_ > 40 kcal mol^−1^), indicating that the release of ring strain in the three- and four-membered systems is an important driving force for the reaction.

**Scheme 4:**
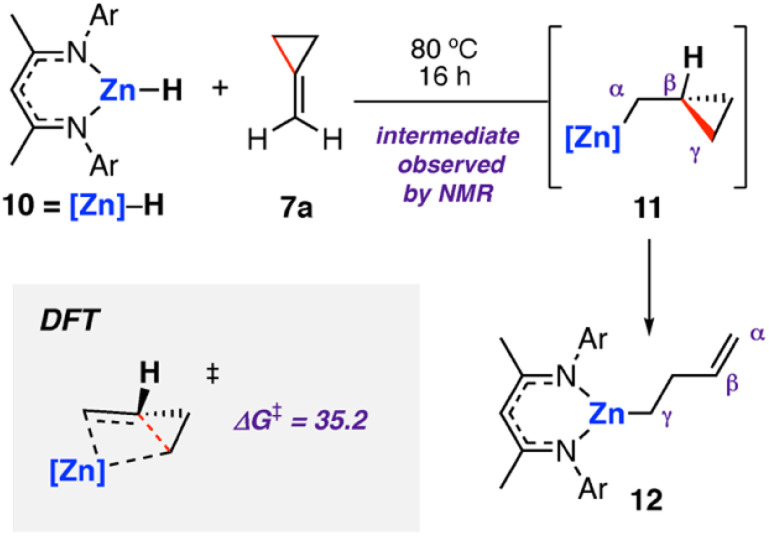
Stoichiometric reaction of zinc hydride complex 10 with methylidene cyclopropane 7a, forming the ring-opened zinc complex 12*via* C–C σ-bond activation at Zn. Ar = 2,6-diisopropylphenyl. Energies for Δ*G*^‡^_298 K_ in kcal mol^−1^.

The analogous zinc hydride complex [ZnH{CH{C(CH_3_)NDipp}_2_}]^53^10 was shown to react with methylidene cyclopropane 7a in a similar fashion.^49^ The resulting zinc alkenyl complex 12 was isolated in high yield and characterised. In the case of zinc, the proposed hydrozincated intermediate 11 was observed spectroscopically (Scheme 4). Diagnostic resonances in the ^1^H NMR spectra at *δ* = −0.39 to −0.36 and *δ* = 0.15 to 0.20 ppm were assigned to 11 through application of HSQC and TOCSY NMR methods. DFT calculations support a stepwise hydrometallation and subsequent β-alkyl elimination pathway. The activation barrier for C–C cleavage was calculated as Δ*G*^‡^_298 K_ = 35.2 kcal mol^−1^, in line with the high temperature conditions required for the reaction.

Activation strain analysis^54,55^ was used to explain the differences in reactivity between the analogous zinc and magnesium hydride complexes. Namely, that magnesium was observed to ring open cyclobutane rings, whereas zinc was not. The more electropositive metal (Mg) was shown to be better able to stabilize the hydrocarbon fragment at the C–C activation transition state, lowering the kinetic barrier relative to Zn.^49^6 and 10 have an identical ligand coordination, as such it can be concluded that chemoselectivity in these reactions can be controlled through choice of the metal.

**Scheme 5:**
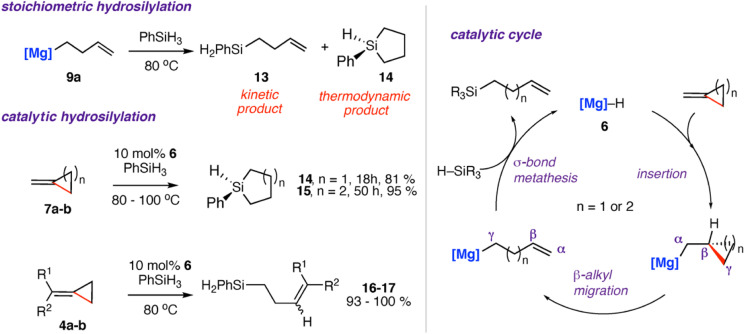
Stoichiometric and catalytic hydrosilylation of alkylidene cycloalkanes 4a–b, 7a–b using a molecular magnesium hydride reagent 6 (left). Proposed catalytic cycle (right). [Mg] = [Mg{CH{C(CH_3_)NDipp}_2_}], Dipp = 2,6-diisopropylphenyl. 16, R^1^ = R^2^ = Ph; 17, R^1^ = Ph, R^2^ = H.

Reaction of the magnesium alkenyl complex 9a with an excess of phenyl silane (PhSiH_3_) led to formation of the linear and cyclic silane compounds 13–14 and reformation of magnesium hydride 6. A catalytic protocol for the hydrosilylation of strained σ-C–C bonds was developed from these findings.^42,49,56,57^ Reaction of 4a–b and 7a–b with excess phenyl silane and 10 mol% of 6 showed high conversion to the respective cyclic silanes 14–15. In the case of 4b, catalytic hydrosilylation yields a mixture of *E* and *Z* stereoisomers of the products 16–17, with a ratio of *E* : *Z* of 1 : 1.1. Scheme 5 shows the proposed catalytic cycle, each step of which is supported by experimental and computational data. The stepwise process follows: (i) hydromagnesiation of the alkene through a 1,2-insertion reaction of the magnesium hydride to the alkene (ii) β-alkyl migration; (iii) σ-bond metathesis to regenerate magnesium hydride catalyst 6 and the linear silane. The thermodynamic products 14–15 are likely formed from an intramolecular hydrosilylation also catalysed by the magnesium complex 6. The related zinc complex 10 was unable to catalyse the hydrosilylation of the σ-C–C bond of 7a, again highlighting the divergent reactivity of main-group and post-transition metals in these systems.^49^

The most likely origin of these differences is the σ-bond metathesis step of the proposed catalytic cycle which while operating with Mg, is likely too slow to facilitate turnover with Zn.

### 2.2 Oxidative addition at aluminium centres

Oxidative addition, and its microscopic reverse, reductive elimination, are some of the most fundamental transformations in organometallic chemistry. Biphenylene has been the subject of detailed study in transition-metal mediated C–C σ-bond activation *via* oxidative addition. Recent examples have extended study of the substrate to low-valent aluminium complexes. Biphenylene comprises two benzene rings fused by a central C_4_ ring. The central C–C σ-bond of the four-membered has a low bond dissociation energy of 65.4 kcal mol^−1^.^58,59^ Both the anti-aromatic character and strain of the four-membered ring contribute to the weakening of this C–C σ-bond. In contrast, the C–C bond strength within the six-membered ring system has been estimated as 114.4 kcal mol^−1^. Addition of transition metals to biphenylene results exclusively in σ-C–C bond activation *via* oxidative addition at the central C_4_ ring (M = Fe, Co, Ni, Ru, Rh, Pd, Os, Ir, Pt, Au).^60–65^ Very recently, selective cleavage of the C–C bonds in biphenylene during potassium reduction of rare-earth metal complexes (Sc, Lu) was reported.^59^

**Scheme 6:**
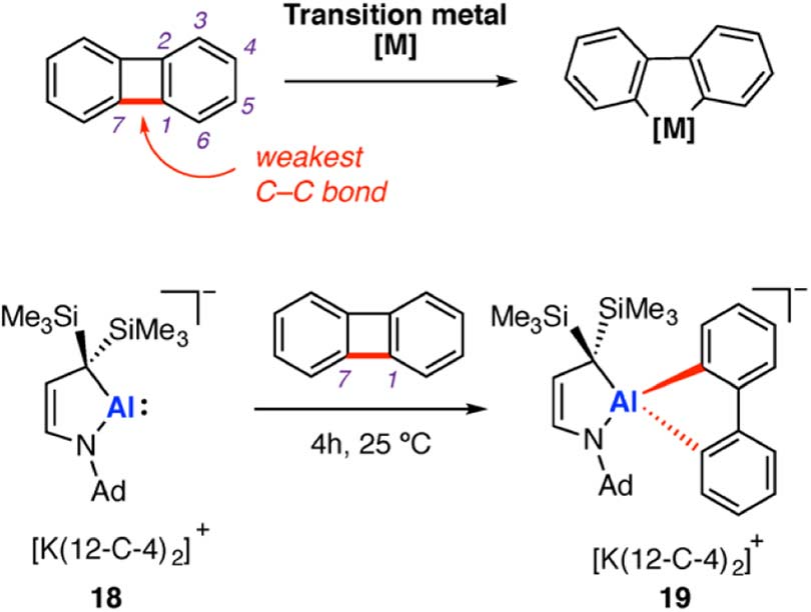
Labelled biphenylene substrate and reported reactivity with transition metals (top); reaction of 18 with biphenylene, resulting in C–C σ-bond scission *via* oxidative addition of the central C^1^–C^7^ σ-bond (bottom). Ad = 1-adamantyl.

In 2020, Kinjo and co-workers reported an aluminyl anion stabilised by a cyclic (alkyl)(amino) ligand, prepared by potassium graphite reduction of the corresponding aluminium dimer in the presence of 12-crown-4.^66^ The resulting aluminyl anion 18 [Al{NAd(CH)_2_C(SiMe_3_)_2_}][K(12-C-4)_2_] was shown to react with biphenylene at room temperature over the course of four hours (Ad = 1-adamantyl, 12-C-4 = 12-crown-4). Oxidative addition of the weakest C–C σ-bond in biphenylene (in the strained four membered ring) was observed (Scheme 6). The metallocyclic anionic complex 19 was isolated in moderate yield (33%) and crystallographically characterised. The overall reaction can be categorised as a two-electron oxidative addition reaction at Al, from Al(i) to Al(iii). Oxidative addition of the central four membered ring of biphenylene is well known for most transition metals, with this work extending the concept to main-group metal complexes for the first time. DFT calculations, performed by Zhu and co-workers, suggest C–C σ-bond activation of benzene by 18 could become both kinetically and thermodynamically favourable through addition of electron withdrawing groups onto benzene.^67^ In practice though, inclusion of additional functionality raises the issue of chemoselectivity; most of these functional groups would contain C–X (X = heteroatom) bonds that are likely to react in preference to the C–C bond.

### 2.3 C–C bond activation initiated by cycloadditions

**Scheme 7:**
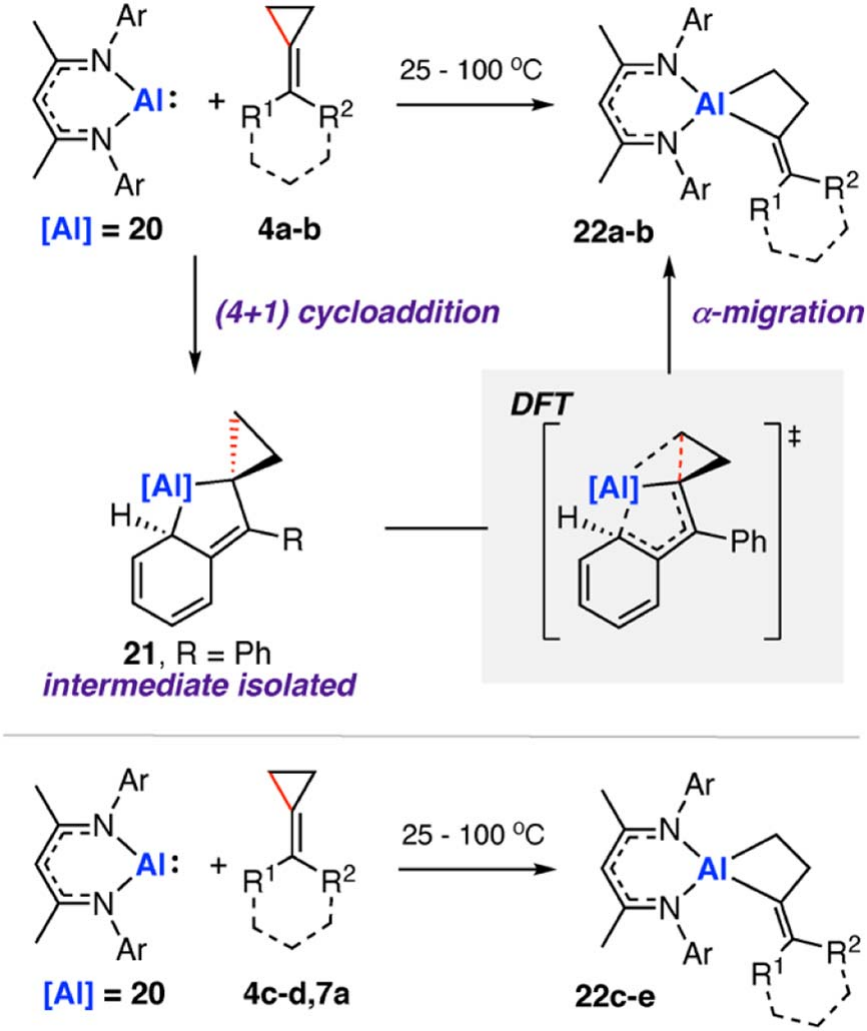
Reaction of aluminium(i) nucleophile 20 with a series of methylidene cycloalkanes 4a, R^1^ = R^2^ = Ph; 4b R^1^ = Ph, R^2^ = H; 4c R^1^ = R^2^ = –(CH_2_)_5_–; 4d R^1^ = Cy, R^2^ = H; 7a R^1^ = R^2^ = H; 22a R^1^ = R^2^ = Ph; 22b R^1^ = Ph, R^2^ = H; 22c R^1^ = R^2^ = –(CH_2_)_5_–; 22d R^1^ = Cy, R^2^ = H; 22e R^1^ = R^2^ = H).

In 2020, we reported the reaction of a β-diketiminate stabilised aluminium(i) nucleophile^71^20 with a series of unsaturated cyclopropanes 4a–d, 7a (Scheme 7). Take 1,1′-(cyclopropylidenemethylene)dibenzene (4a, R^1^ = R^2^ = Ph) as a representative example. Reaction of 20 with 4a initially formed an Al(iii) metallocyclopentane complex 21*via* a (4 + 1) cycloaddition. DFT calculations show a low energy barrier (Δ*G*^‡^_298 K_ = 14.1 kcal mol^−1^) for the exergonic formation of 21 from 20 and 4a
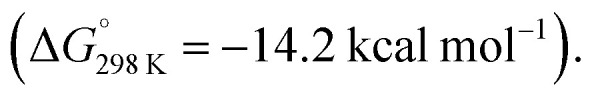
 Heating crude or isolated samples of 21 resulted in C–C σ-bond activation of the cyclopropane ring and formation of metallocyclobutane complex 22. The reaction proceeded rapidly at 100 °C (<15 min) or slowly (over a few days) at ambient temperature. Relief of ring strain provides a thermodynamic driving force for the overall reaction. DFT calculations suggest an α-migration pathway connecting intermediate 21 with the C–C activated product 22 (Δ*G*^‡^_298 K_ = 25.8 kcal mol^−1^). The scope was extended to four other alkylidene cyclopropanes, showing a related α-migration pathway for C–C σ-bond activation at the Al(i) centre. For methylidene cyclopropanes with alkyl substituents, 4c–d, reaction with 20 is presumed to occur through an initial (2 + 1) cycloaddition intermediate, as the (4 + 1) cycloaddition becomes inaccessible. For comparison, the direct oxidative addition of a C–C σ-bond in 4a by 20 was calculated to proceed *via* a considerably higher energy transition state (Δ*G*^‡^_298 K_ = 35.3 kcal mol^−1^). The data suggests the key factor for C–C σ-bond scission is not dependent on a redox reaction at the aluminium centre, rather on the installation of the electropositive Al atom in the correct position on the hydrocarbon scaffold to facilitate the α-alkyl migration rearrangement.

**Scheme 8:**
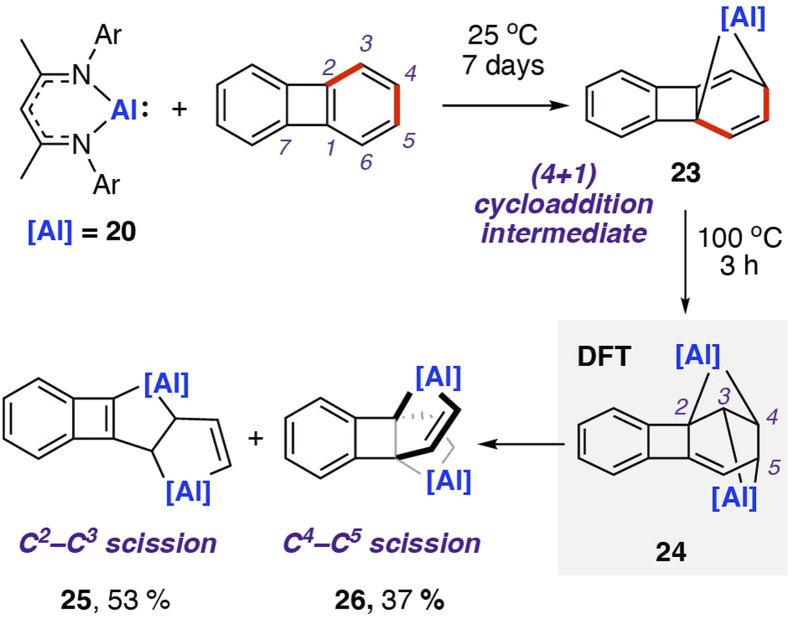
Reaction of aluminum(i) nucleophile 20 with biphenylene, giving a mixture of products formed *via* C–C σ-bond cleavage. Ar = 2,6-diisopropylphenyl; NMR yield shown for product mixture.

In 2021, our group reported the reaction of 20 with biphenylene.^75^ This was the first report of chemoselective C–C bond activation of biphenylene, and a rare example where the substrate bias is overcome by reagent control. Reaction of two equivalents of 20 with biphenylene and heating to 100 °C yielded a mixture of metallocyclic complexes 25 and 26 in which aluminium(iii) centres are incorporated in five-membered rings (Scheme 8). 25 and 26 are derived from the cleavage of the C^2^–C^3^ and C^4^–C^5^ bonds in the C_6_ ring of biphenylene, respectively. These complexes can be separated. Heating purified samples of either product showed that they did not interconvert under the reaction conditions.

DFT calculations suggest an initial (4 + 1) cycloaddition reaction between 20 with biphenylene,^76^ specifically a [_π_4_s_ + _n_2_s_] cycloaddition, yielding a highly strained and dearomatised hydrocarbon scaffold. The intermediate 23 can be isolated from the stoichiometric reaction of 20 with biphenylene at 25 °C for seven days. From 23, DFT calculations suggest a pathway for addition of a second equivalent of 20 to the C_2_–C_3_ position and a subsequent concerted rearrangement to the more thermodynamically favourable isomer 24. The isomerisation is likely driven by the interchange of the three- and five-membered rings within the first intermediate to two four-membered rings in the second intermediate, relieving ring strain in the system. From 24, two similar energy pathways to products 25 and 26 are proposed, *via* either C–C bond cleavage and subsequent 1,3-sigmatropic shift, or a 1,3-sigmatropic shift followed by C–C bond cleavage. Both pathways are highly exergonic, consistent with the nonreversible formation of the products observed experimentally. A direct oxidative addition of the central C^1^–C^7^ σ-bond of biphenylene to 20 was calculated to occur by a high activation barrier (Δ*G*^‡^_298 K_ = 42.0 kcal mol^−1^), likely to be inaccessible under the reaction conditions. Further calculations using activation strain analysis suggest that the inaccessible energy barriers for oxidative addition are likely a result of the strain required to achieve orbital overlap between the aluminium complex's lone pair and C^1^–C^7^ σ*-orbital in biphenylene.

In 2022, Liu and co-workers reported the synthesis of an N-heterocyclic carbene (NHC) stabilised aluminylene compound, featuring a bulky carbazolyl ligand 27.^72–74^ DFT calculations show a decreased HOMO–LUMO gap upon NHC coordination to 27 compared with the analogue without the additional coordinated ligand. 27 was shown to reversibly convert into the aluminocyclic complex 28 formed by an intramolecular insertion of Al into the flanking 3,5-di-*tert*-butylphenyl rings of 27. DFT calculations support a reversible process 
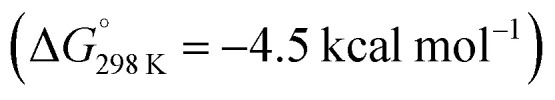
*via* a concerted transition state (Δ*G*^‡^_298 K_ = 25.6 kcal mol^−1^) to form the AlC_6_ ring. Calculations for the analogous pathway without NHC coordination showed a kinetically unfavourable activation barrier of Δ*G*^‡^_298 K_ > 70 kcal mol^−1^.

**Scheme 9:**
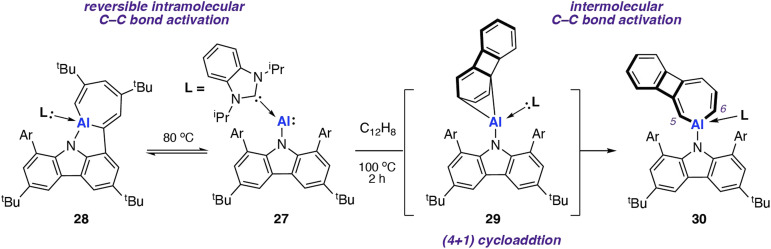
Intra- (left) and inter-(right) C–C bond activation with NHC coordinated aluminylene 27. Ar = 3,5-di-*tert*-butylphenyl.

Onward reaction of 27 with biphenylene at 100 °C resulted in the formation of the AlC_6_ aluminocyclic complex 30 now derived from an intermolecular pathway (Scheme 9). Aromatic C–C bond scission occurred with the central (and weakest) C_4_ unit remaining intact. This is the only report for the C–C bond cleavage of the C^5^–C^6^ bond in biphenylene for any metal. A (4 + 1) cycloaddition intermediate 29 was isolated from the reaction of 27 and biphenylene at 25 °C; a related (4 + 1) cycloaddition product was observed from reaction of 27 and naphthalene. DFT calculations suggest that C–C bond breaking can occur at intermediate 29, albeit through a high energy transition state (Δ*G*^‡^_298 K_ = 37.9 kcal mol^−1^). As this barrier is beyond what would be predicted for a process operating at 100 °C, alternative mechanisms for C–C bond, including bimetallic pathways, remain a possibility.

**Scheme 10:**
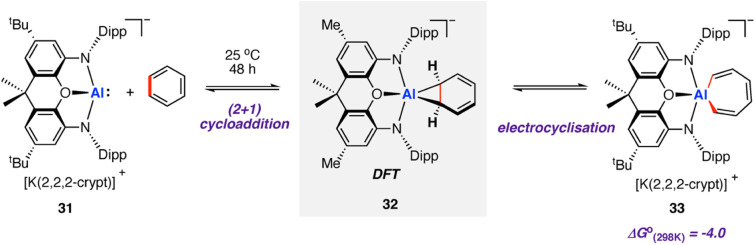
Reversible insertion of aluminium(i) complex 31 into the C–C bond of benzene.

In 2019, Aldridge, Goicoechea, and co-workers reported the reversible room temperature C–C bond activation of benzene using a nucleophilic aluminium complex (Scheme 10).^68^ Reaction of [K(2.2.2-crypt)][(NON)Al] (31, where NON = 4,5-bis(2,6-diisopropyl-anilido)-2,7-di-*tert*-butyl-9,9-dimethylxanthene) with benzene at room temperature gave near quantitative formation of the corresponding aluminium(iii) cycloheptatriene 33. A cross-over experiment in which deuterium labelling C_6_D_6_ was added to 31 showed that the reaction is reversible, as evidenced by formation d^6^-33 and C_6_H_6_
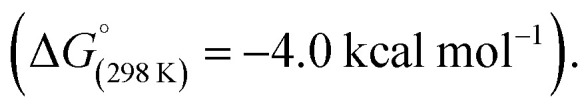
 Addition of Me_2_SnCl_2_ to 33 generated the *Z*,*Z*,*Z*-isomer of the dimetallated heptatriene, Me_2_ClSnCH=CH–CH=CH–CH=CHSnClMe_2_ (34), derived from benzene. The aluminocycle 33 formed by C–C bond activation was shown to be a kinetic product that formed reversibly. When samples were heated to 80 °C non-reversible C–H activation of the benzene ring was observed. A subsequent computational investigation by Fernández and co-workers explored the competing C–C and C–H bond activation of benzene by 31 using a combination of activation strain and energy decomposition analyses.^69^ Calculations support C–C bond activation as a kinetic pathway, as observed experimentally. The overall oxidative addition occurs through a stepwise mechanism, initiated by a (2 + 1)^70^ cycloaddition reaction to form an aluminocyclopropane intermediate 32. A 6π electrocyclic ring-opening of the aluminocyclopropane 32, formally a Büchner ring expansion, cleaves the C–C bond to give 33 in an exergonic process 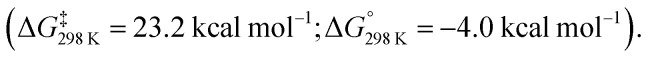 Reaction of 31 with naphthalene gave the C–H activation product only.

**Scheme 11:**
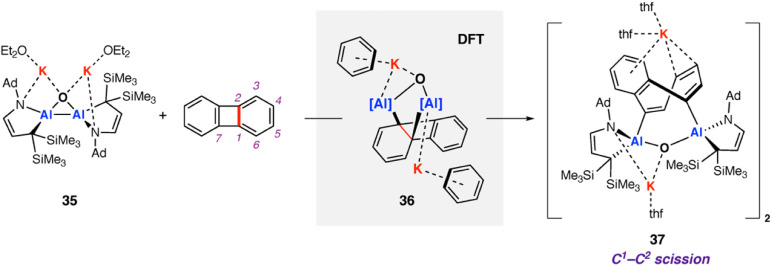
Reaction of dianionic dialane complex 35 with biphenylene. C^1^–C^2^ activation of biphenylene observed in the product 37. Note: line-drawing for 37 has been represented as in the original publication.

In 2021, Kinjo and co-workers reported a dianionic dialane complex 35 featuring an Al_2_O three-membered ring supported by two K^+^ ions. 35 was formed by the reduction of the corresponding dialane and subsequent treatment with triethylphosphine oxide.^77^ Onward reaction of 35 with a series of small molecules, including biphenylene, was investigated (Scheme 11). Stoichiometric reaction of 35 with biphenylene at room temperature yielded a colourless precipitate (37) isolable in moderate (31%) yield. Multinuclear NMR spectroscopy and X-ray diffraction analysis showed the dimeric product 37, formed by unusual C–C bond cleavage of the C^1^–C^2^ bond in biphenylene. DFT calculations were employed to gain insight into a likely reaction mechanism. Coordination of biphenylene to potassium antedates Al–Al bond cleavage and Al–C bond formation. The initial reaction step involves coordination of 35 to form the biphenylene adduct. Two subsequent 1,3-sigmatropic rearrangements result in migration of the Al centre across the hydrocarbon scaffold to form 36 (Δ*G*^‡^_298 K_ = 19.9 then 25.8 kcal mol^−1^). 36 contains a five-membered AlOC_2_Al ring, primed for C–C bond activation. C–C bond activation proceeds *via* a low energy transition state (Δ*G*^‡^_298 K_ = 3.4 kcal mol^−1^) to give the observed product 37. The overall process from 35 and biphenylene to 37 is exergonic 
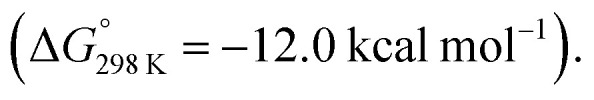
 The K^+^ ions were modelled explicitly in this pathway and may play an important role in structural organization and facilitating C–C bond activation.

**Scheme 12:**
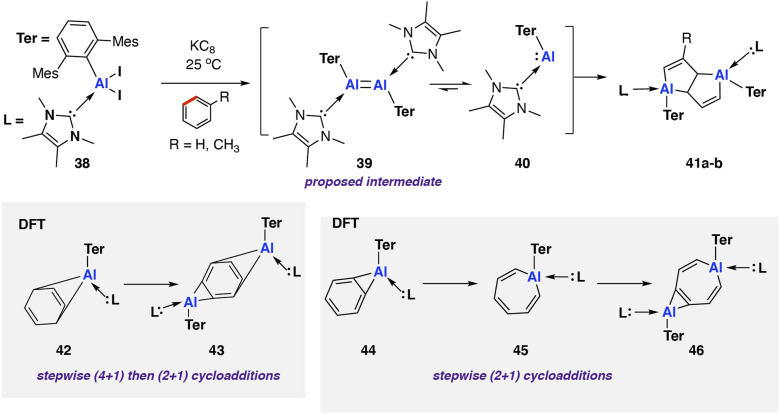
C–C bond scission of benzene and toluene by an *in situ* generated NHC-coordinated aluminylene 41 (Mes = 2,4,6-trimethylphenyl; 42a R = H; 42b R = CH_3_).

In 2022, Braunschweig and co-workers reported an *in situ* generated base-stabilised aryl aluminene (*viz.* aluminylene) complex capable of deconstructing benzene and toluene *via* C–C bond scission (Scheme 12).^78^ The reaction was very low yielding toward the C–C activation product (3–5% isolated yield) with the major product formed *via* an intramolecular C–H activation of the mesityl ligand. Reduction of the parent NHC-coordinated aluminium diiodo complex [Al(NHC)Ar*(I)_2_] (NHC = 1,3,4,5-tetramethylimidazol-2-ylidene; Ar* = 2,6-C_6_H_3_Mes_2_, Mes = 2,4,6-trimethylphenyl) 38 with KC_8_ yielded a mixture of C–H and C–C 41a–b activation products. The reaction mechanism was proposed to proceed *via* formation of di-aluminene complex [{Al(NHC)Ar*}_2_] 39 and subsequent monomerisation to the reactive aluminium(i) nucleophile [Al(NHC)Ar*] 40. As these species are generated *in situ*, however, the speciation of the aluminium complexes and role of K^+^ remains ambiguous.

Coordination of the NHC ligand was essential to increase the reactivity of the aluminium centre, facilitating the observed C–C bond activation. A related base free aluminene was reported previously and showed no reactivity toward aromatic solvents.^79^ DFT calculations suggest that the frontier molecular orbitals of 40 are aluminium-based. Coordination of the NHC ligand decreases the HOMO–LUMO gap from 3.82 eV to 3.23 eV, with an increase in the HOMO energy the major contribution to the change.

Further calculations were performed to investigate a likely reaction mechanism to form pentalene complex 41a; for computational cost, a truncated analog of 40 was used [Al(NHC′)Ar′] (NHC′ = 1,5-dimethylimidazol-2-ylidene; Ar′ = 2,6-C_6_H_3_Xyl_2_, Xyl = 2,6-dimethylphenyl). Two potential mechanisms were investigated computationally. The authors comment that both pathways are feasible with low and reversible energy barriers for C–C bond activation and are likely competing. The first pathway, analogous to the reaction of 20 with biphenylene,^75^ proceeds *via* a (4 + 1) cycloaddition by the aluminylene with benzene, followed by a (2 + 1) cycloaddition from another equivalent of 40 on the opposite face of the benzene molecule to create a bimetallic complex 43. Rearrangement and concomitant C–C bond activation of the benzene unit yields 41a in an overall exergonic process 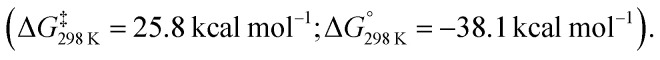


The second pathway involves an initial formal insertion of aluminylene 40 into the C–C bond of benzene *via* an initial (2 + 1) cycloaddition forming an aluminocyclopropane intermediate 44. Reaction of a second equivalent of 40 with this intermediate again facilitates C–C bond cleavage, yielding the observed product 41a (Δ*G*^‡^_298 K_ = 17.4 kcal mol^−1^).

## 3. Conclusions and perspective

Carbon–carbon bond activation facilitated by main-group and post-transition metal complexes is gathering increasing attention. The above summary of known examples of this type of reactivity shows that main-group metal complexes can mimic, and complement, reactivity long associated with late transition metals. Common to several of these systems is the idea that the key factor is the ability to install the electropositive and coordinatively unsaturated metal atom *e.g.* Mg, Al, or Zn, in a suitable position on a hydrocarbon framework to facilitate migration/rearrangement reactions. Surprisingly redox based processes, *e.g.* oxidative addition, are not often invoked in the actual C–C bond breaking step, this contrasts examples with transition metals where they are common. The divergence in behaviour may well reflect the spatial availability of the orbitals involved in reactivity, with main group and post-transition metal complexes typically characterised by spatially orthogonal HOMO and LUMO orbitals with large energy separation, while transition metals often have a manifold of multiple available d-orbitals, which (in combination) are more spatially flexible.

In this regard, it is notable that many of the emerging applications of low-valent aluminium in C–C bond activation rely on low-oxidation state complexes with coordinated ligands (*e.g.* NHCs). The coordination event not only lowers the HOMO–LUMO gap it also changes the geometry at the metal centre. Both may be important in reaching accessible transition states for C–C bond activation that might not otherwise be possible with the ligand-free counterparts. Similarly, cooperative effects between two or more metals offer alternative pathways to break C–C bonds with reagents that are constrained to a certain set of orbital interactions. Bimetallic pathways have been invoked in several of the systems known to date, with two metals acting in concert; binding, distorting, and destabilising hydrocarbon frameworks to achieve C–C bond activation.

In the immediate future, it is likely that new and interesting examples of C–C bond activation with main group and post-transition metals will be discovered. Investigation of low-valent aluminium reagents appears to be a particularly fertile area. Systematic studies that aim to generate an understanding of why examples of C–C bond activation with certain metals are so prevalent, and what factors influence reactivity (*e.g.* coordination geometry, orbital energies, orbital symmetry, electronegativity of M, M–C bond dissociation energies) will help the field develop. Only a small number of main group and post-transition metal elements have been reported to facilitate C–C bond activation. Investigation of complexes of electrophilic main group metals such as those of Ca, Sr, Ba, Ga, In, Sn, and Pb is warranted and may bring with it new mechanistic insight and/or opportunities to control selectivity by tuning the metal site. The scope of reactivity described so far spans some of the most activated strained cycloalkanes and least reactive aromatic carbon rings. There is enormous opportunity in the middle ground. Expansion of examples of C–C bond activation to medium and large cycloalkane rings along with fragmentation of branched and linear alkane chains are obvious targets for the field.

Catalytic protocols for C–C bond functionalisation *e.g.* through hydrosilylation are beginning to emerge. There is a clear need for development of new catalysts for C–C bond functionalisation. The ability to alter hydrocarbon scaffolds of complex organic molecules and to valourise simple hydrocarbons or aromatics through catalysis are particularly attractive approaches that have long been associated with late transition metals. In the longer term, such catalytic transformations could underpin sustainable chemical manufacturing practices including the valourisation of molecules from biomass or the recycling of hydrocarbon-based polymers. The efforts described above suggest that main group systems have the potential to make important contributions in these areas that complement transition metal systems while also addressing key aspects of element scarcity, supply chain risk, and sustainability.

## Author contributions

Both authors contributed to the writing of this manuscript.

## Conflicts of interest

There are no conflicts to declare.

## Supplementary Material
